# Dynamic Injection and Permutation Coding for Enhanced Data Transmission

**DOI:** 10.3390/e26080685

**Published:** 2024-08-13

**Authors:** Kehinde Ogunyanda, Opeyemi O. Ogunyanda, Thokozani Shongwe

**Affiliations:** 1 BT Group, London E1 8EE, UK; 2 Center for Telecommunications, Department of Electrical and Electronic Engineering Science, University of Johannesburg, Johannesburg 2006, South Africa; opeyemiogunyanda@gmail.com; 3 Department of Electrical and Electronic Engineering Technology, University of Johannesburg, Johannesburg 2094, South Africa; tshongwe@uj.ac.za

**Keywords:** adaptive coding, coding with injections, permutation coding, power line communications, spectral efficiency

## Abstract

In this paper, we propose a novel approach to enhance spectral efficiency in communication systems by dynamically adjusting the mapping between cyclic permutation coding (CPC) and its injected form. By monitoring channel conditions such as interference levels and impulsive noise strength, the system optimises the coding scheme to maximise data transmission reliability and efficiency. The CPC method employed in this work maps information bits onto non-binary symbols in a cyclic manner, aiming to improve the Hamming distance between mapped symbols. To address challenges such as low data rates inherent in permutation coding, injection techniques are introduced by removing δ column(s) from the CPC codebook. Comparative analyses demonstrate that the proposed dynamic adaptation scheme outperforms conventional permutation coding and injection schemes. Additionally, we present a generalised mathematical expression to describe the relationship between the spectral efficiencies of both coding schemes. This dynamic approach ensures efficient and reliable communication in environments with varying levels of interference and impulsive noise, highlighting its potential applicability to systems like power line communications.

## 1. Introduction

Efficient and reliable data transmission is crucial across various communication systems, particularly in environments characterised by noise, jamming and fading [1]. Efforts to enhance communication systems have included the introduction of channel coding techniques, which mathematically manipulate the streams of 0’s and 1’s present in the digital information.

Various types of coding schemes with different complexities, applications and capabilities have been reported in the literature. Examples include Reed Solomon (RS) codes, convolutional codes and permutation coding (PC). RS codes are widely employed in combating burst errors, which occur for a continuous duration of time in the communication channel [1,2,3]. PC methods, including cyclic permutation coding (CPC), have been demonstrated to be effective in combating channel impairments like narrowband interference and impulsive noise, which are issues in power line communications (PLCs) [4,5,6]. However, the resulting low coding rate remains a challenge.

Injection coding is a variant of PC which offers higher symbol rates and larger cardinality, making it an attractive alternative to ordinary PC [7,8]. In this paper, we therefore introduce a novel approach to enhance the data transmission performance of any PC-based system by introducing dynamic adaptation of injection and CPC techniques. This allows the coding strategy to be tailored to channel conditions like background noise, impulsive noise and interference, resulting in enhanced error correction and spectral efficiency.

The primary contribution of this work lies in the development and evaluation of this adaptive coding scheme. We provide a comparative analysis with traditional CPC methods to demonstrate the efficacy of our approach. We also develop a mathematical formulation and a proposition statement to elucidate the relationship between the spectral efficiencies inherent in both coding schemes. Furthermore, the technique focuses on complementing PC’s low data rate, thereby corroborating its usefulness in various applications like PLC.

While we discuss the potential application of our coding scheme to PLC, the focus is on the broader applicability and benefits of the proposed method. Hence, this work promotes the use of adaptive PC techniques to address challenges posed by impulsive noise and interference in various communication systems, not limited to PLC. Other applications include channels with memory where error bursts are common [9,10,11], flash memory, and digital recording, where permutation coding has been previously proposed for error correction and spectral shaping [12,13].

The rest of this paper is organised as follows. Section 2 looks into CPC, providing a detailed explanation of its characteristics, and then introduces injection coding, highlighting its advantages over PC. Section 3 provides a detailed analysis comparing both approaches in terms of coding rate, which translates to spectral efficiency. Section 4 outlines our proposed dynamic adaptation scheme and its algorithm. Moving on to Section 5, we present the simulation and the channel models employed for evaluating the proposed dynamic adaptation scheme. In Section 6, we showcase results comparing the proposed scheme with conventional CPC, analyse performance metrics such as bit error rate under different channel conditions, and interpret the results and their implications. Finally, in Section 7, we offer our conclusion, summarising the key findings of the study and proposing future research directions.

## 2. Permutation Coding (PC) and Injection Coding

The basic idea behind permutation coding is to rearrange the symbols representing the information bits in a systematic manner, which can help improve the robustness of the transmission against channel disturbances. By selecting appropriate permutations, permutation coding can enhance properties such as error correction capability and resistance to specific types of noise [14,15].

PC is made up of codewords $C_{\tau }$ with non-repetitive symbols from the set Q={0,1,…,p−1} [14,16,17]. Here, *p* is the symbol size and τ=1,2,…,|C|, with |C| being the cardinality that indicates the maximum number of codewords in the codebook C.

Here is an example permutation mapping:

(1) $$\overset{\text{data bits}}{\underset{\text{PC symbols}}{\left\{\begin{array}{cccc} 00 & 10 & 01 & 11\\ ↓ & ↓ & ↓ & ↓\\ 3120 & 1203 & 2031 & 0312 \end{array}\right\},}}$$

where k=2 data bits are mapped onto M=4 PC symbols.

The minimum Hamming distance $H_{\min}$, which is the least possible distance between any two codewords, can be used to define a codebook’s cardinality |C| as [16]:

(2) $$\left|C\right|\leq \frac{M!}{(H_{\min}-1)!}.$$

### 2.1. Cyclic Permutation Coding (CPC)

CPC is a specific type of permutation coding where the symbols are arranged cyclically [6], meaning that the order of symbols is shifted by a fixed amount for each permutation. The codebook in (1) is a typical example of a CPC. The 2nd codeword is derived from the 1st one by shifting the 1st symbol to the back. The 3rd and 4th codewords are derived in a similar approach. This cyclic arrangement provides certain benefits in terms of error correction, noise resilience and data synchronisation.

Given a PC codebook with codeword length *M*, symbol size *p* and cardinality |C|, the coding rate *R* is given by

(3) $$R=\frac{\log_{2}\left|C\right|}{M\log_{2}p}=\frac{k}{m}.$$

The numerator of (3) is the number of *k* data bits mapped to *M* PC symbols, while the denominator is the bit equivalent (i.e., *m*) of the PC symbols in the codebook. In PCs, *M* is the same as *p*.

If k<m, it then implies that the codebook introduces redundant bits *r*, which can be defined by

(4) $$r=M\log_{2}p-\log_{2}\left|C\right|.$$

### 2.2. Cyclic Permutation Coding with Injections

Considering the minimum Hamming distance $H_{\min}$, a codebook can detect up to *t* errors as per the expression given by [15]:

(5) $$t\leq H_{\min}-1,$$

where *t* denotes the maximum number of detectable errors. Additionally, the codebook can correct up to t/2 errors. Therefore, enhancing the error-correcting capability of a system is feasible by increasing its $H_{\min}$. However, such improvement comes at the expense of reducing the coding rate *R* [18]. A conventional approach to increase *R* is reducing the $H_{\min}$ and the *p* while increasing the |C|. However, by introducing injections in the original PC codebook, these sacrifices can be leveraged. This means the *p* and the |C| can be fixed while reducing the $H_{\min}$ to attain higher *R*. This is best illustrated by deleting the last column of the CPC in (1), resulting in

(6) $$\begin{array}{c} \left\{\begin{array}{c} 3\;1\;2\;0\\ 1\;2\;0\;3\\ 2\;0\;3\;1\\ 0\;3\;1\;2 \end{array}\right\}\;\to \;\left\{\begin{array}{c} 3\;1\;2\\ 1\;2\;0\\ 2\;0\;3\\ 0\;3\;1 \end{array}\right\}. \end{array}$$

This means that δ=1 column is deleted in the source PC. Therefore, given that $C_{\tau }=\left\{c_{0},c_{1},\ldots ,c_{p-1}\right\}$, $C_{\tau }^{\prime }\subset C_{\tau }$ and $Q^{\prime }\subset Q$, with the length of $Q^{\prime }$ being $M^{\prime }$, then an $M^{\prime }$ arrangement of $C_{\tau }$ is an injection of $C_{\tau }^{\prime }$ into $C_{\tau }$ if $M\geq M^{\prime }$. As such, PC codewords are derived from the permutations of all the symbols in *Q*, while subsets of *Q* are permuted to obtain injection codewords [8,9].

Upon reviewing the concepts of code puncturing as outlined in the literature [7], one might draw parallels between puncturing and injection. Nevertheless, in the context of permutation coding, we adhere to the terminology established in the existing PC literature [8,9], wherein the process is consistently referred to as injections.

## 3. Rate Gain Analysis: CPC vs. Injected CPC

Going forward, we shall use notations with the superscript ${(}^{\prime })$ to denote any variable related to injection codebooks, and those without ${(}^{\prime })$ for ordinary PCs. The two codebooks in (6) are thus characterised by M=4, $M^{\prime }=3$, $H_{\min}=4$, $H_{\min}^{\prime }=3$, respectively, while $\left|C|=\right|C^{\prime }|=4$ and $p=p^{\prime }=4$.

Using (3) and (4), we find that

(7) $$\begin{array}{cc} \; & R=\frac{\log_{2}\left|C\right|}{M\log_{2}p}=\frac{\log_{2}4}{4\log_{2}4}=\frac{1}{4},\\ \; & R^{\prime }=\frac{\log_{2}\left|C^{\prime }\right|}{M^{\prime }\log_{2}p^{\prime }}=\frac{\log_{2}4}{3\log_{2}4}=\frac{1}{3} \end{array}$$

and

(8) $$\begin{array}{cc} \; & r=M\log_{2}p-\log_{2}\left|C\right|=4\log_{2}4-\log_{2}4=6,\\ \; & r^{\prime }=M^{\prime }\log_{2}p^{\prime }-\log_{2}\left|C^{\prime }\right|=3\log_{2}p-\log_{2}4=4. \end{array}$$

Depending on the design objectives, it is possible to delete δ>1 columns in the source PC to yield the target injection codebook with much higher $R^{\prime }$. It is also possible to maintain $H_{\min}=H_{\min}^{\prime }$, and $M=M^{\prime }$, thereby achieving $|C^{\prime }\left|>|C\right|$, which in turn gives $R^{\prime }>R$. This is possible by making $p^{\prime }>p$. This is, however, beyond the scope of this work.

**Proposition** **1.** *Given that a target injected CPC is derived from a source CPC by deleting δ column(s), the rate gain ($R_{g}$) between the two codebooks is expressed as*

(9) $$R_{g}=\frac{\delta }{M^{2}-M\delta }\times 100\%,$$

*where M is the codeword length of the source CPC.*

**Proof** **of Proposition 1.** If *R* and $R^{\prime }$ represent the rates of the CPC and its injected form, respectively, the increase in the rate, in %, can be said to be

(10) $$R_{g}=\frac{R^{\prime }-R}{R}\times 100\%.$$

By substituting the expression in (3) in the above, we have

$$R_{g}=\frac{\left(\frac{\log_{2}\left|C^{\prime }\right|}{M^{\prime }\log_{2}p^{\prime }}\right)-\left(\frac{\log_{2}\left|C\right|}{M\log_{2}p}\right)}{\left(\frac{\log_{2}\left|C\right|}{M\log_{2}p}\right)}\times 100\%.$$

If $\left|C|=\right|C^{\prime }|$ and $p=p^{\prime }$, we further simplify the expression to obtain

$$\begin{array}{cc} R_{g} & =\frac{\log_{2}\left|C\right|\left(\frac{1}{M^{\prime }\log_{2}p}-\frac{1}{M\log_{2}p}\right)}{\log_{2}\left|C\right|\times \frac{1}{M\log_{2}p}}\times 100\%\\ \; & =\left(\frac{1}{M^{\prime }\log_{2}p}-\frac{1}{M\log_{2}p}\right)\times \frac{M\log_{2}p}{1}\times 100\%. \end{array}$$

Further factorisation reduces the expression to

(11) $$R_{g}=\left(\frac{1}{M^{\prime }}-\frac{1}{M}\right)\times 100\%.$$

Given that δ is the number of the injected column(s) in the CPC to obtain the target injected form, it then means that $M^{\prime }=M-\delta$. If this is substituted in (11), we have

(12) $$\begin{array}{cc} R_{g} & =\left(\frac{1}{M-\delta }-\frac{1}{M}\right)\times 100\%,\\ \; & =\left(\frac{M-\left(M-\delta \right)}{M\left(M-\delta \right)}\right)\times 100\%, \end{array}$$

which, when further factorised, yields the expression in (9) as the proposition states.    □

**Figure 1:**
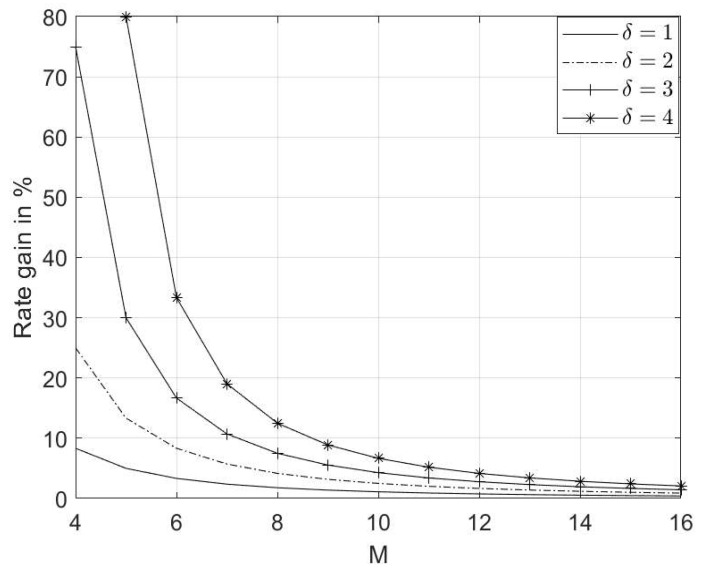
Rate gain vs. *M* at various δ values.

Rate gains for different scenarios of δ, with $\left|C|=\right|C^{\prime }|$, are depicted in Figure 1. As δ increases, the rate gain increases. Also, if δ is fixed, the $R_{g}$ becomes insignificant as *M* increases. However, it is important to note that the advantage of injection coding becomes more significant when a greater value of δ is injected, especially at higher levels of *M*.

## 4. CPC-Injected CPC Dynamic Adaptation

Several dynamic adaptation schemes have been proposed in the literature, focusing on either coding or modulation schemes. For instance, some schemes utilise channel estimators such as the minimum-mean-square-error channel estimator to adaptively adjust transmission parameters based on channel conditions [19]. Others leverage metrics like the Block Error Rate (BLER) derived from channel feedback to dynamically modify transmission strategies, such as adjusting transmission repetitions [20]. In [21], the metrics used are signal-to-noise ratio (SNR) and correlation coefficient.

**Figure 2:**
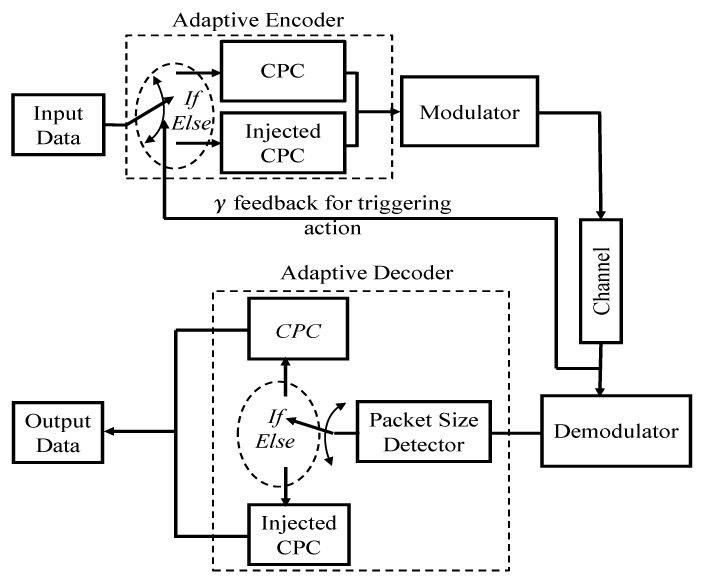
Logical topology for dynamic adaptation.

In our proposed adaptive coding scheme shown in Figure 2, the mode of operation revolves around threshold decisions derived from channel interference levels. These thresholds dynamically determine whether to utilise the CPC or the injected CPC variant. The decision-making process is based on the premise that higher interference levels favour the adoption of more robust coding schemes. As far as we are aware, this is the first time a dynamic adaptation of CPC and its injected form is reported in the literature.

### 4.1. Algorithm for Dynamic Adaptation

The dynamic adaptation scheme is governed by the following Algorithm 1:
**Algorithm 1** Dynamic Adaptation1:Measure channel interference level γ.2:Compare γ with predefined thresholds.3:**If** γ exceeds the thresholds, switch from injected CPC to CPC.4:**Otherwise**, continue with injected CPC.

The selection of the threshold can be probabilistically derived using the BLER [22,23]. Since the probability of bit error depends on the SNR and γ, let η, $P_{e}$, and $P_{b}$ represent the spectral efficiency, bit error probability, and block error probability for CPC, respectively, and let $\eta^{\prime }$, $P_{e}^{\prime }$, and $P_{b}^{\prime }$ represent the corresponding metrics for injected CPC.

The $P_{e}$ can be modelled as a function of the SNR and γ:

$$P_{e}=f\left(\mathrm{SNR},\gamma \right).$$

For injected CPC, the error probability is

$$P_{e}^{\prime }=f^{\prime }\left(\mathrm{SNR},\gamma \right).$$

The block error probability $P_{b}$ can be derived from the bit error probability $P_{e}$ as

$$P_{b}=1-{\left(1-P_{e}\right)}^{N_{s}},$$

where $N_{s}$ is the number of symbols per block [22]. For injected CPC, the block error probability $P_{b}^{\prime }$ is

$$P_{b}^{\prime }=1-{\left(1-P_{e}^{\prime }\right)}^{N_{s}}.$$

To determine the threshold *T*, we define it such that the system switches from injected CPC to CPC when γ exceeds *T*. Hence, *T* is derived by equating the performance metrics of both coding schemes:

(13) $$\eta^{\prime }\cdot \left(1-P_{b}^{\prime }\right)=\eta \cdot \left(1-P_{b}\right).$$

In this context, η is proportional to the coding rate *R*, and thus *T* is the value of γ for which this equation holds, balancing the spectral efficiencies of both schemes. This, therefore, infers that the adaptive switching threshold is reached whenever the performances of both CPC and injected CPC cross paths or are relatively close to each other.

### 4.2. Pseudocode for the Dynamic Adaptation Algorithm

Based on the algorithm presented in Section 4.1, Pseudocode 1 below can be followed for reproducibility.
**Pseudocode 1** Dynamic Adaptation**Require:** SNR, $N_{s}$ (number of symbols per block)**Ensure:** Selected coding scheme (CPC or injected CPC)1:Default to injected CPC2:Measure the channel interference level, γ3:Calculate bit error probabilities:4:$P_{e}^{\prime }=f^{\prime }\left(\mathrm{SNR},\gamma \right)$, $P_{e}=f\left(\mathrm{SNR},\gamma \right)$5:Calculate block error probabilities:6:$P_{b}^{\prime }=1-{\left(1-P_{e}^{\prime }\right)}^{N_{s}}$, $P_{b}=1-{\left(1-P_{e}\right)}^{N_{s}}$7:Determine the threshold *T* by equating the performance metrics:8:$\eta^{\prime }\cdot \left(1-P_{b}^{\prime }\right)=\eta \cdot \left(1-P_{b}\right)$9:Compare γ with the threshold *T*10:**if** γ exceeds *T* **then**11:   Switch to CPC12:**end if**

## 5. Channel Simulation Model

In this work, we adopt a model utilising a Gaussian random variable to simulate background noise inherent in communication channels as additive white Gaussian noise (AWGN). This model typically assumes a constant noise power spectral density (PSD) across all frequencies and a fixed variance for the Gaussian noise.

If *n* represents Gaussian noise with zero mean and constant variance $\sigma^{2}$, it is sampled from a Gaussian distribution with a probability density function given by

(14) $$p\left(n\right)=\frac{1}{\sqrt{2\pi \sigma^{2}}}\exp\left(-\frac{n^{2}}{2\sigma^{2}}\right).$$

In the case of interference noise, no direct relationship is found between its PSD and that of AWGN, as opposed to impulsive noise [24]. The PSD for impulsive noise is flat and covers all frequencies, with variance $\sigma_{I}^{2}$ related to $\sigma^{2}$ as follows [25]:

(15) $$\sigma_{I}^{2}=\frac{\sigma^{2}}{\Gamma },$$

where Γ<1 determines the impulsive noise strength, and when Γ→∞, the impulsive noise is of no significance.

Since interference is unpredictable and its PSD is only flat over a section of the spectrum, we denote the probability of its occurrence as *A*. Hence, for each given *A* and Γ, we take the PSD strength of the interference noise as γ=1/(AΓ) [24]. As such, γ is dependent on cases where Γ<1.

To simulate interference in our work, we corrupt the received data with a signal of randomly varying amplitude due to its unpredictability. This approach more accurately reflects the nature of interference in practical communication systems, ensuring the robustness and relevance of the simulation results. The simulated noise model was developed based on the channel models reported in [24,25].

Various switching thresholds of γ, influenced by 1/(AΓ), are explored to observe the combined effects of interference noise and impulsive noise. The parameter γ influences the amplitude of the interference.

The adaptive coding scheme transitions from the default injection coding (with a higher rate $R^{\prime }$) to a more robust CPC (with a lower rate *R*) once γ surpasses the thresholds. For fair comparisons, coding rates are enforced in all simulations conducted.

## 6. Results and Discussion

Here, we present the simulation results obtained when the proposed adaptive coding scheme is compared with the conventional CPC and its injected variant. We denote the ordinary CPC-coded transmission as the CPC scheme. The case where the data are coded with only an injected CPC codebook is termed the injection scheme, and the case where the adaptive switching between the CPC and its injected form are involved is termed the adaptive scheme. An uncoded QPSK scheme is also simulated alongside the the evaluated schemes for the purpose of validating the simulation work.

**Figure 3:**
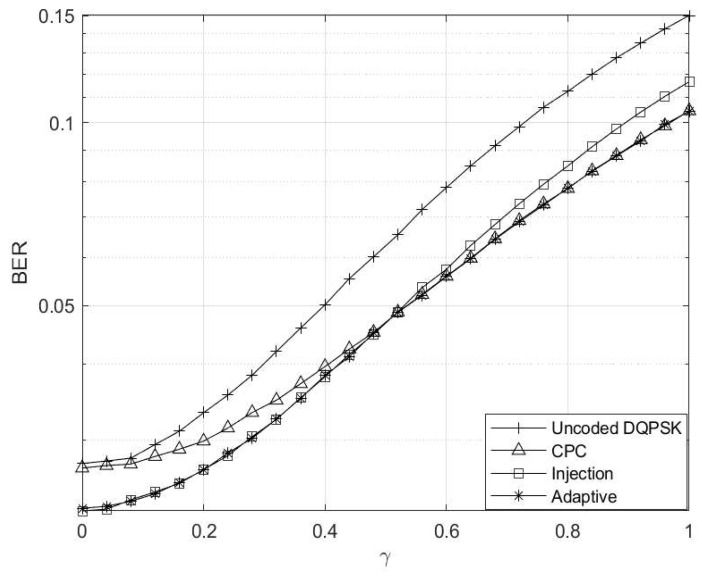
Interference level γ vs. BER for M=4 and $M^{\prime }=3$ codebooks.

The analysis of the simulation results depicted in Figure 3 illustrates a notable trend wherein error rates increase with elevated interference levels across all schemes. Notably, the uncoded DQPSK scheme demonstrates inferior performance, while the injection scheme emerges as the best and preferred scheme at γ≤0.5. However, as γ surpasses 0.5, the CPC scheme emerges as the top performer. This behaviour prompts the adaptive scheme to transition from injection coding to CPC once γ reaches or exceeds 0.5. This threshold was only chosen solely for the purpose of analyses.

**Figure 4:**
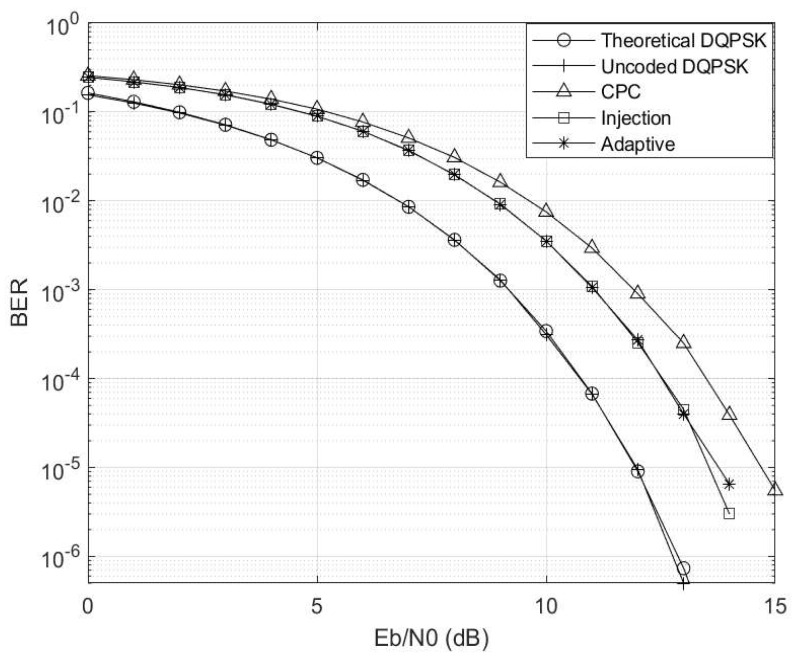
$E_{b}/N_{0}$ vs. BER at γ=0 and Γ→∞ for M=4 and $M^{\prime }=3$ codebooks.

The $E_{b}/N_{0}$ is varied while making γ=0 and Γ→∞, and the corresponding results are depicted in Figure 4. Here, the setup mirrors that of a conventional AWGN channel. Notably, in the absence of any channel interference and impulsive noise, the uncoded scheme outperforms the coded schemes due to coding rate compensation. Furthermore, the injection coding scheme outperforms the CPC scheme, aligning with the region where γ≤0.5, consistent with the observations in Figure 3. Additionally, it is worth highlighting that the adaptive scheme closely tracks the performance trajectory of the injection scheme, as the threshold condition of γ≥0.5 has not yet been met.

**Figure 5:**
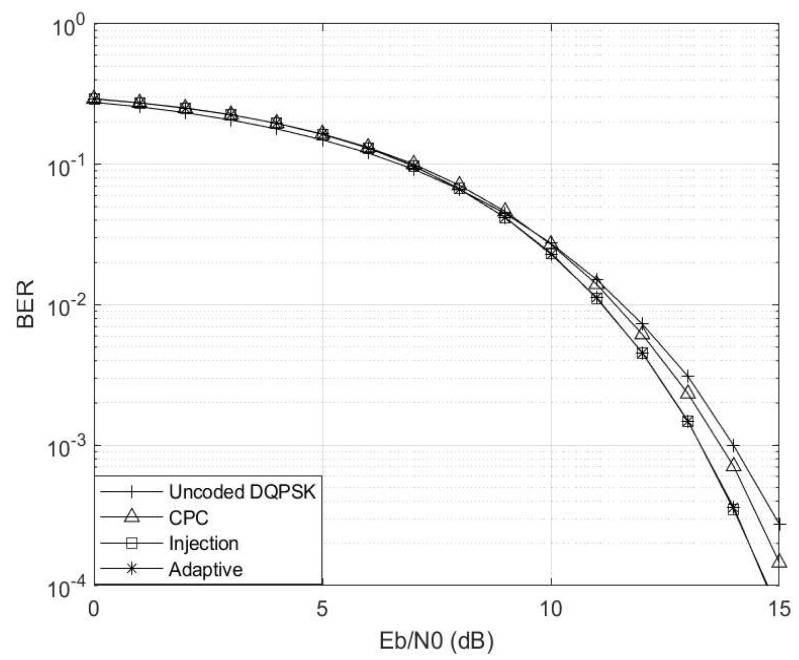
$E_{b}/N_{0}$ vs. BER at γ=0.01 and Γ=0.99 for M=4 and $M^{\prime }=3$ codebooks.

Figure 5 showcases the simulation outcomes with γ=0.01 and Γ=0.99. At $E_{b}/N_{0}\geq 9$ dB, the uncoded scheme lags behind the coded schemes. Since the dynamic switching threshold is set at γ≥0.5, no switching occurs under these circumstances. Consequently, the adaptive scheme consistently mirrors the behaviour of the injection coding scheme throughout this scenario. It is worth noting that in this instance, the CPC scheme still exhibits slightly inferior performance compared to the injection scheme, underscoring the importance of embracing the proposed adaptive scheme.

**Figure 6:**
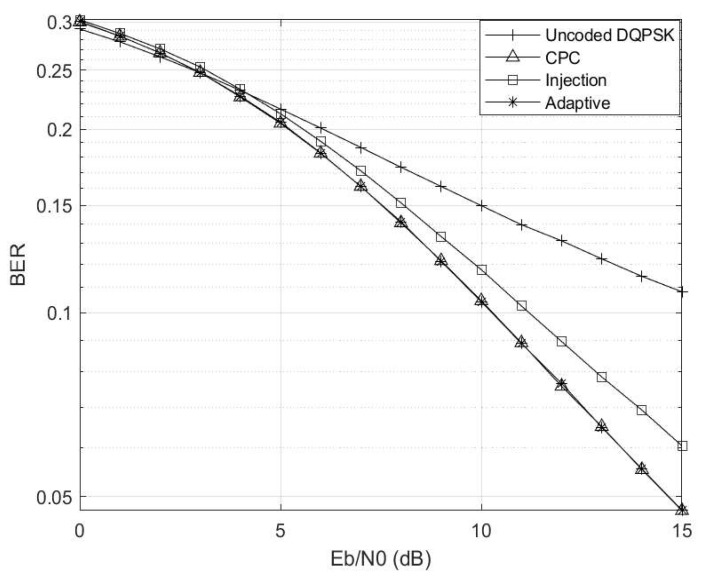
$E_{b}/N_{0}$ vs. BER at γ=1 and Γ=0.99 for M=4 and $M^{\prime }=3$ codebooks.

Fixing γ and Γ at 1 and 0.99, respectively, we obtain the results shown in Figure 6. At $E_{b}/N_{0}>5$ dB, the uncoded scheme becomes adversely affected by the channel interference and is unable to compete the coded schemes. The adaptive scheme is seen to follow the best-performing CPC scheme because the switching threshold is exceeded.

We extended the simulation exercises to evaluate an M=8 CPC and its $M^{\prime }=3$ injected equivalent (i.e., δ=5). Figure 7, Figure 8, Figure 9 and Figure 10 show the results obtained, which are similar to those in Figure 3, Figure 4, Figure 5 and Figure 6.

All the simulations conducted reveal the pronounced significance of the CPC scheme particularly at elevated γ levels. Consequently, the adaptive scheme effectively capitalises on the injection coding’s high-rate capability during instances of lower γ. Additionally, it is noteworthy that while evaluating coded communications schemes, substantial enhancements over the uncoded scheme are anticipated, especially when considering channel conditions beyond mere channel interference and impulsive noise, with particular interest in lower $E_{b}/N_{0}$ values.

Overall, the outstanding performance of the adaptive coding scheme is much more significant when compared with the uncoded scheme and the coded schemes (i.e., the CPC and the injected CPC). This is due to a better spectral efficiency achieved through a better coding rate, when the dynamic coding scheme switches to the injected CPC codebook at γ values below the switching threshold. Further improvement is expected when the the coding scheme is cascaded with another coding scheme such as convolutional coding (CC) or Reed Solomon (RS) code. According to the literature, a PC scheme, precoded with RS code, has been demonstrated to exhibit similar behaviour to that of an RS-precoded CC scheme [24]. Consequently, in [6], the CPC scheme was shown to be superior to its CC and PC counterparts in combating various PLC-related noise when precoded with RS code. By introducing injections into the CPC scheme in this work, we have observed improved performance, especially when used adaptively with the CPC. This further supports the use of the PC scheme for combating PLC-related impairments on a larger scale. As such, the proposed scheme can be regarded as an extension of the work reported in [6].

## 7. Conclusions

This work reported a dynamic adaptation scheme aimed at enhancing spectral efficiency in communication systems. By dynamically adjusting the permutation mapping between cyclic permutation coding (CPC) and its injected form based on channel conditions, the system optimises data transmission reliability and efficiency. Comparative analyses show that the adaptive scheme outperforms conventional permutation coding and injection schemes, with the injection scheme showing promise for enhanced performance where channel interference is mild, particularly concerning coding rate compensation.

All the schemes evaluated exhibit increasing error rates with higher interference levels, with the adaptive scheme effectively transitioning between CPC and injection coding at appropriate thresholds. At higher $E_{b}/N_{0}$ levels, both the CPC and adaptive schemes consistently outperform the uncoded QPSK scheme, emphasising the effectiveness of coding techniques for error mitigation. Additionally, Proposition 1, which provides a mathematical expression for the rate gain between CPC and injected CPC, offers insights into the potential spectral efficiency gains achievable through injection coding. Overall, the proposed dynamic coding approach holds significant potential for enhancing spectral efficiency in diverse communications environments, particularly when combined with additional coding schemes. Further research and experimentation are needed to fully explore and implement this dynamic coding paradigm in real-world communications systems like the PLC where other forms of channel impairments are present.

## Figures and Tables

**Figure 7:**
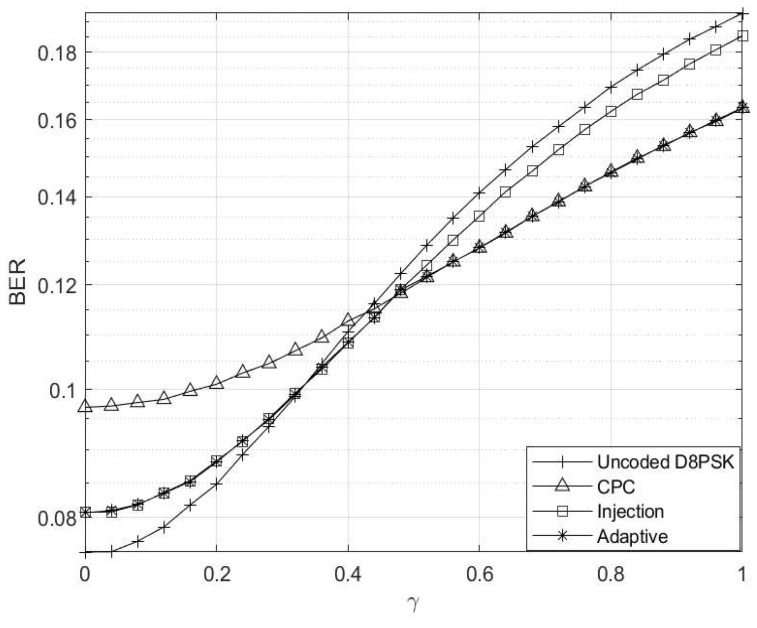
Interference level γ vs. BER for M=8 and $M^{\prime }=3$ codebooks.

**Figure 8:**
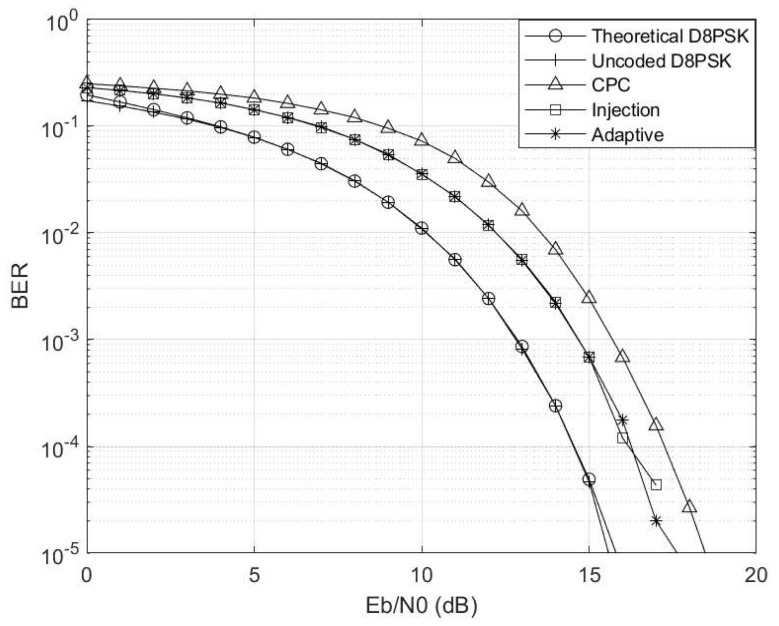
$E_{b}/N_{0}$ vs. BER at γ=0 and Γ→∞ for M=8 and $M^{\prime }=3$ codebooks.

**Figure 9:**
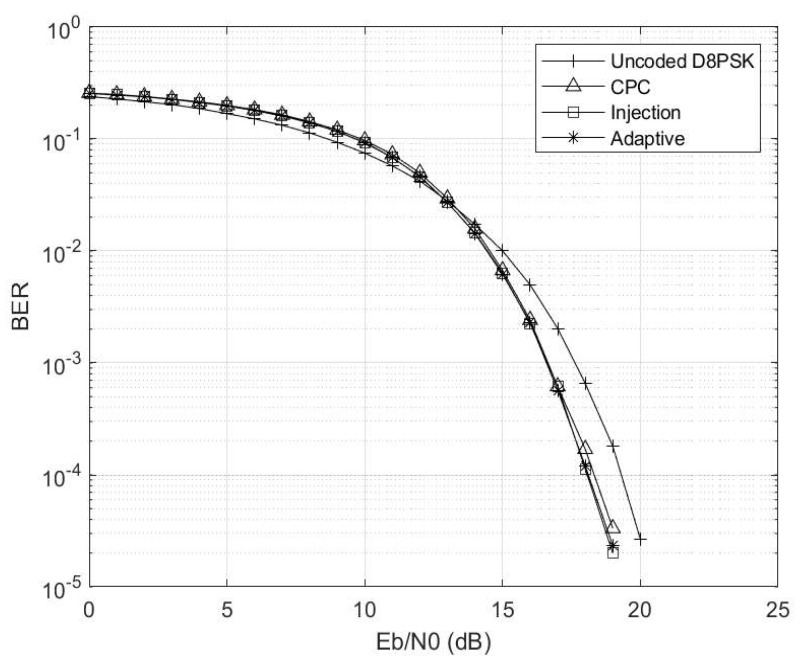
$E_{b}/N_{0}$ vs. BER at γ=0.01 and Γ=0.99 for M=8 and $M^{\prime }=3$ codebooks.

**Figure 10:**
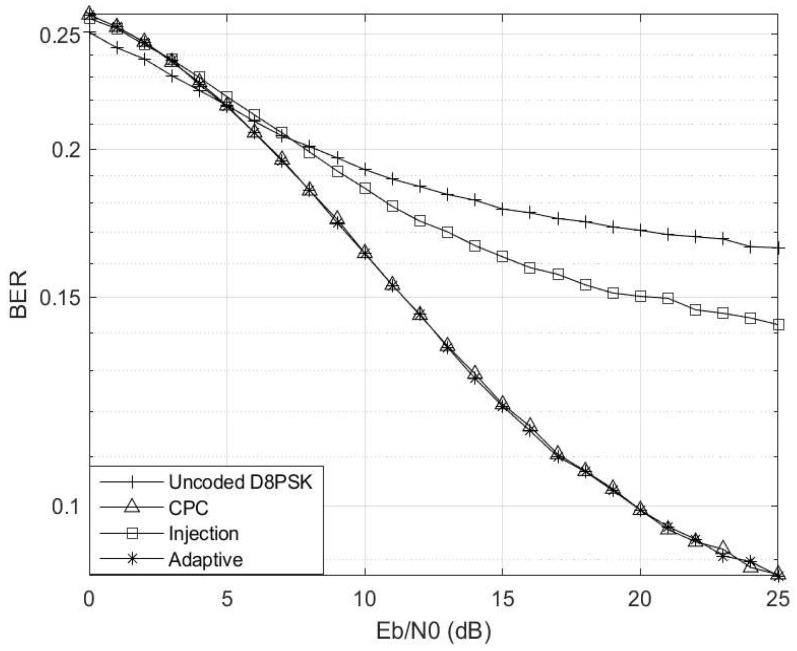
$E_{b}/N_{0}$ vs. BER at γ=1 and Γ=0.99 for M=8 and $M^{\prime }=3$ codebooks.

## Data Availability

The original contributions presented in the study are included in the article, further inquiries can be directed to the corresponding author.
